# I SPY WITH MY LITTLE EYE: WHAT VISUAL SEARCH CAN TELL US ABOUT HOW WE SEE THE WORLD

**DOI:** 10.3389/frym.2019.00004

**Published:** 2019-01-28

**Authors:** Allison Joanna Lewis, Isabella Noel Nemer, Jay Hegdé

**Affiliations:** 1 Department of Biology, College of Science and Mathematics, Augusta University, Augusta, GA, United States; 2 Department of Neuroscience and Regenerative Medicine, James and Jean Culver Vision Discovery Institute, Augusta, GA, United States; 3 Department of Ophthalmology, Medical College of Georgia, Augusta University, Augusta, GA, United States

## Abstract

We have all have experienced the frustration of looking for something we want, only to find a seemingly endless series of things we do not want. This process of looking for an object of interest is called visual search. We perform visual search all the time in everyday life, because the objects we want are almost always surrounded by many other objects. But, in some cases, it takes special training to find things, such as when searching for cancers in X-rays, weapons or explosives in airport luggage, or an enemy sniper hidden in the bushes. Understanding how we search for, and find, objects we are looking for is crucial to understanding how ordinary people and experts alike operate in the real world. While much remains to be discovered, what we have learned so far offers a fascinating window into how we see.

## VISUAL SEARCH IS A FACT OF LIFE, AND CAN BE A MATTER OF LIFE OR DEATH

**Figure 1:**
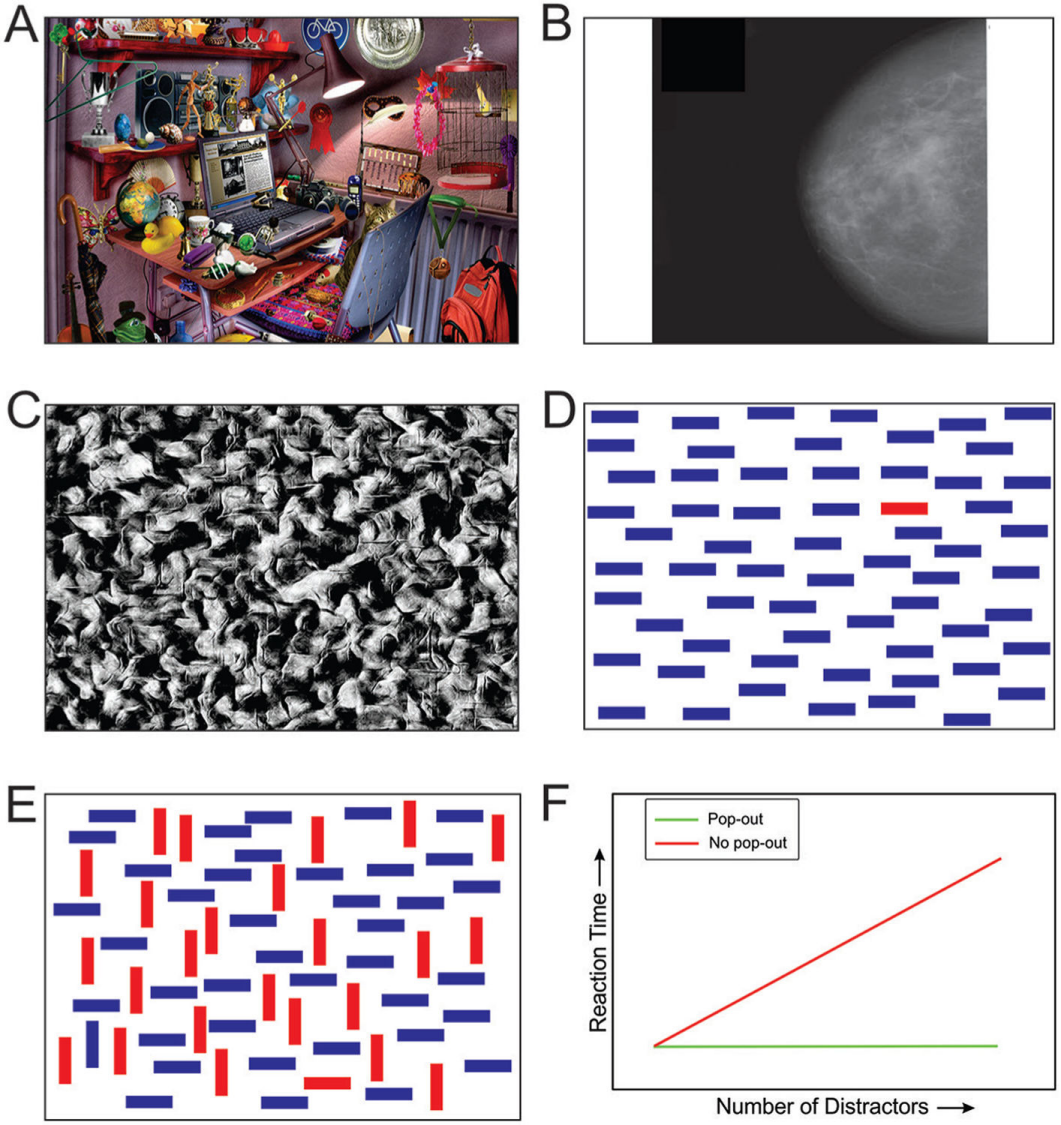
Visual scenes with multiple objects always require visual search. **(A)** A typical “I SPY” scene. Can you find the peanut? (For the answer, see arrow in Figure 3A). **(B)** An x-ray image of a breast with cancer. While the bright parts of the image toward the front of the breast tend to attract our attention, they are not cancerous. Thus, they are simply distractors. The cancerous region is much subtler (see arrow in Figure 3B). It takes considerable medical training and experience to learn to detect such cancers. **(C)** Can you find the human head camouflaged against the background? (For the answer, see Figure 3C). **(D)** A pop-out scene in which the odd-man-out, the horizontal red bar, is easily found (see arrow in Figure 3D). **(E)** Under certain circumstances, including most real-world scenes, the target does not pop out, because it shares some characteristics of the other objects in the scene (see arrow in Figure 3E). **(F)** The graph shows how reaction times for locating a target change depending on the number of distractors in the image and whether the target pops out or not.

Many of us remember passing time on long, dreary road trips by playing “I SPY” (Figure 1A) or “Airport Scanner” on our cell phones or tablets. Older people-such those in their thirties and forties-may remember “Where’s Waldo?” a popular game found in comic books of their day. These activities are fun, because they make a game out of what we do in real life all the time-that is, look for an object of interest, or a “target,” among distracting things that we do not want, or “distractors.” These games let us experience the sweet frustration of looking for a target and not finding it, or the joy of finding it.

Sometimes, finding a target takes special training and can be a matter of life or death. Doctors train for years to become good at finding cancers in breast X-rays (Figure 1B) or chest X-rays, for example. Similarly, military specialists undergo extensive training to find enemy snipers or landmines camouflaged against the background. Disaster can strike if smart machines, such as driverless cars or medical diagnostic equipment, fail to find the targets they are designed to find. In the wild, animals must spot potential predators quickly enough to flee to safety. On the other hand, a predator may starve to death if it is not good enough at finding its prey, or if its prey finds it too easily.

So, you can clearly see that **visual search** is a very important part of how the brain works in real life.

## THE BASICS OF VISUAL SEARCH

How does the brain find what it is looking for, and how do researchers go about understanding how the brain does this? For one thing, researchers often use simplified, computer-generated visual “scenes” (Figures 1C-E). This is because it is easier to create such images in sufficiently large numbers (imagine having to find thousands of real-world images of camouflaged snipers), and because this makes visual search simpler, which in turn makes it easier to study visual search in the laboratory.

Important early studies performed by Anne Treisman (1935–2018), beginning in the 1960s, used simple images to uncover some basic things about visual search [1, 2]. For her contributions to our understanding of visual search, Dr. Treisman was awarded the National Medal of Science by U.S. President Barack Obama in 2013.

Imagine you are told to find the object that does not belong, or the “**odd-man-out**,” in Figure 1D. What you are looking for in this figure, the target, is the red bar. In this case, the target can be easily recognized. The red bar “pops out,” and it does so regardless of whether the distractors are blue bars or some other object, like apples. So, a target pops out if it can be distinguished from all the distractors using a basic visual characteristic, or “feature.” In the above example, this distinguishing feature happens to be color, but the distinguishing feature could also be shape, orientation, motion, depth, etc. Treisman found that the number of distractors does not matter in this case. The target can be found just as easily among four distractors as among 40 distractors. In this case, the time it takes for an observer to find the target, or “reaction time,” does not depend on the number of objects in the scene (green line in Figure 1F).

Reaction times are quite different when the target shares some, but not all, of the features of some of the distractors (Figure 1E). In this case, the target does not pop out (“non-pop-out target”), and the reaction times go up according to the number of objects in the visual scene (red line in Figure 1F).

Why does this happen? What is the simplest explanation for both types of reaction time patterns seen in Figure 1F? A critical insight came when Treisman and colleagues closely examined the errors made by the observers. When the observers misperceived what the target was (for instance, when they reported that the odd-man-out was a red vertical bar, when it actually was a red horizontal bar, Figure 1D), they were also likely to misperceive where the target was. This means that, in order to find a target correctly, one also has to perceive the target correctly. Additional experiments showed that mental ability to focus on a particular location in the visual scene—say, the bar at the top left corner of the image—is needed when the target does not pop out, and not needed when it does. This ability is called “spatial attention.” Treisman also showed that, during visual search for a non-pop-out target, we focus **attention** at a given location to see if that location contains the target, and if it does not, we shift the attention to the next location, and so forth until the target is found. Thus, spatial attention and visual search are closely related.

This model of visual search fully explains the reaction time patterns in Figure 1F. But, this model also explains many of the key features of how we find objects in more complex, real-world scenes [1, 2].

## DIFFERENT REGIONS IN THE BRAIN PLAY DIFFERENT ROLES IN VISUAL SEARCH

The findings described above tell us that visual search must involve communication between the brain regions involved in recognizing an object (“What is it?”), and the regions involved in focusing attention on a given location (“Where is it?”) and shifting attention from one location to the next (“Where to, next?”). We know something about how the brain carries out each of these pieces, although the way they work together in the process of visual search is not entirely clear yet.

**Figure 2:**
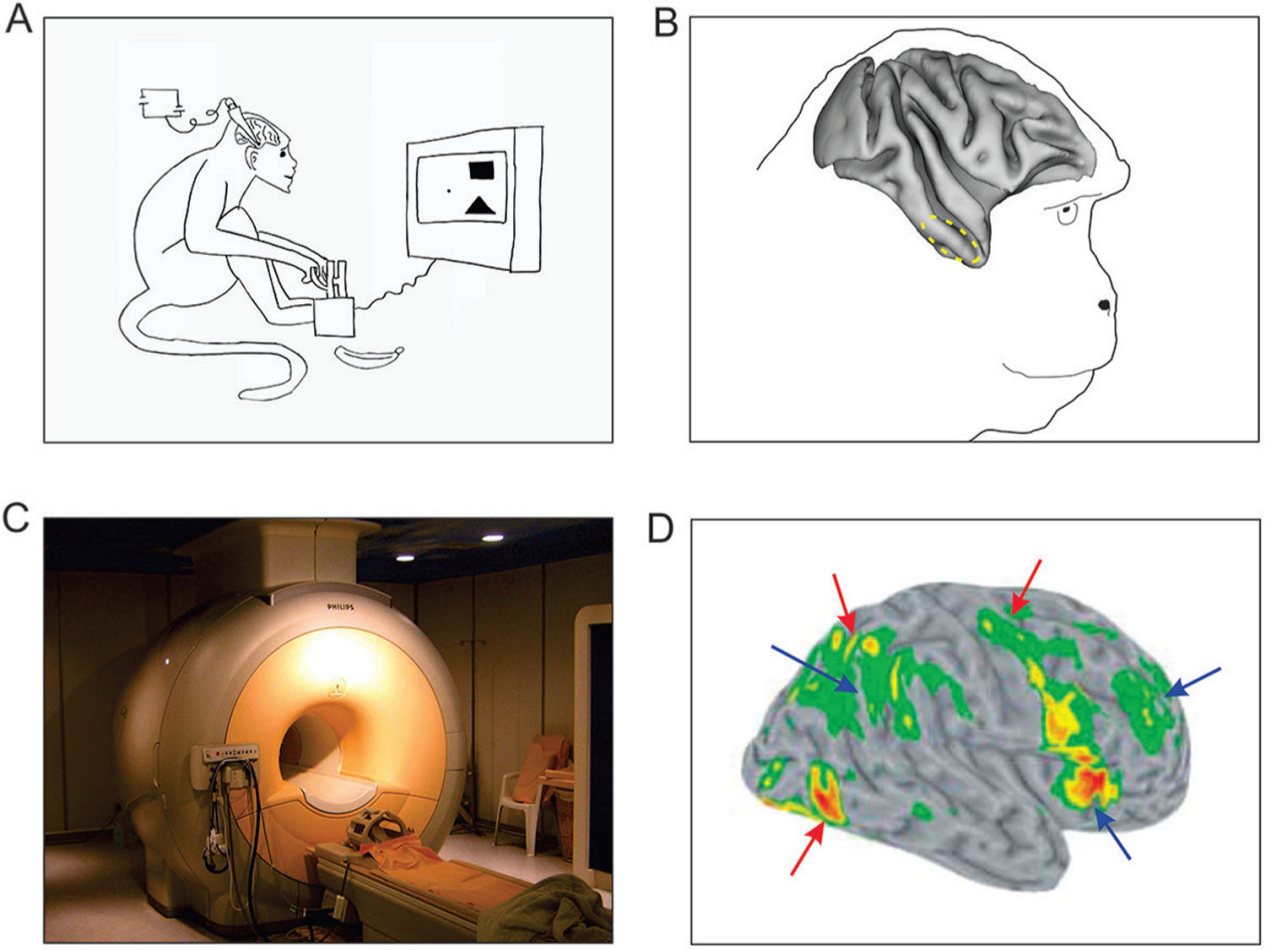
Studying brain responses during visual search. **(A)** A monkey searching for a target (a square or a triangle, depending on the experiment), with a slender wire, called a microelectrode, inserted into the brain to measures the responses of the brain cells in that region. **(B)** The dashed circle shows the part of the monkey brain that is active during visual search [3]. **(C)** A typical fMRI machine, which can be used to measure brain activity in humans. **(D)** Brain responses of human observers during a visual search task [4], measured using fMRI. Colored areas show brain regions that are especially active during the task. The “hotter” colors indicate higher activity. Red arrows show the brain regions that are involved in searching for the target. Blue arrows show the brain regions that are involved when the target is detected, or shortly thereafter. Together, these regions are a part of a brain network that plays a role in focusing and shifting attention.

In 1993, Robert Desimone and colleagues used long, ultra-thin wires inserted into the brains of macaque monkeys, to study how the brain responds while an animal performs a visual search task (Figures 2A, B). They found some brain cells that respond differently depending on which one of the two targets the animal was searching for [3]. Since monkey and human brains are so much alike, it is not surprising that very similar processes have since been found to occur in human brains during visual search, as well.

In human subjects, of course, scientists cannot introduce wires into the brain. Much of what we know about how the human brain performs visual search comes from a technique called **functional magnetic resonance imaging** (fMRI; Figure 2C). fMRI measures the brain activity indirectly, by looking at the changes in blood flow in the brain that happen when brain cells become active. Many fMRI studies have examined how the brain regions involved in attention respond when humans perform visual search. As expected from the earlier studies we have described different brain regions are indeed specialized for various parts of visual search [4]. For instance, some regions of the brain are preferentially active during the visual search (red arrows in Figure 2D), suggesting that they are specialized for shifting attention from one search location to the next. Some other regions of the brain are active when the subject finds the target, suggesting that these regions might help with focusing attention on the object when the subject is looking at one specific search location. How these various brain regions work together to bring about visual search and target detection remains to be understood.

## DAMAGE TO CERTAIN BRAIN REGIONS CAN MAKE VISUAL SEARCH MORE DIFFICULT

**Figure 3:**
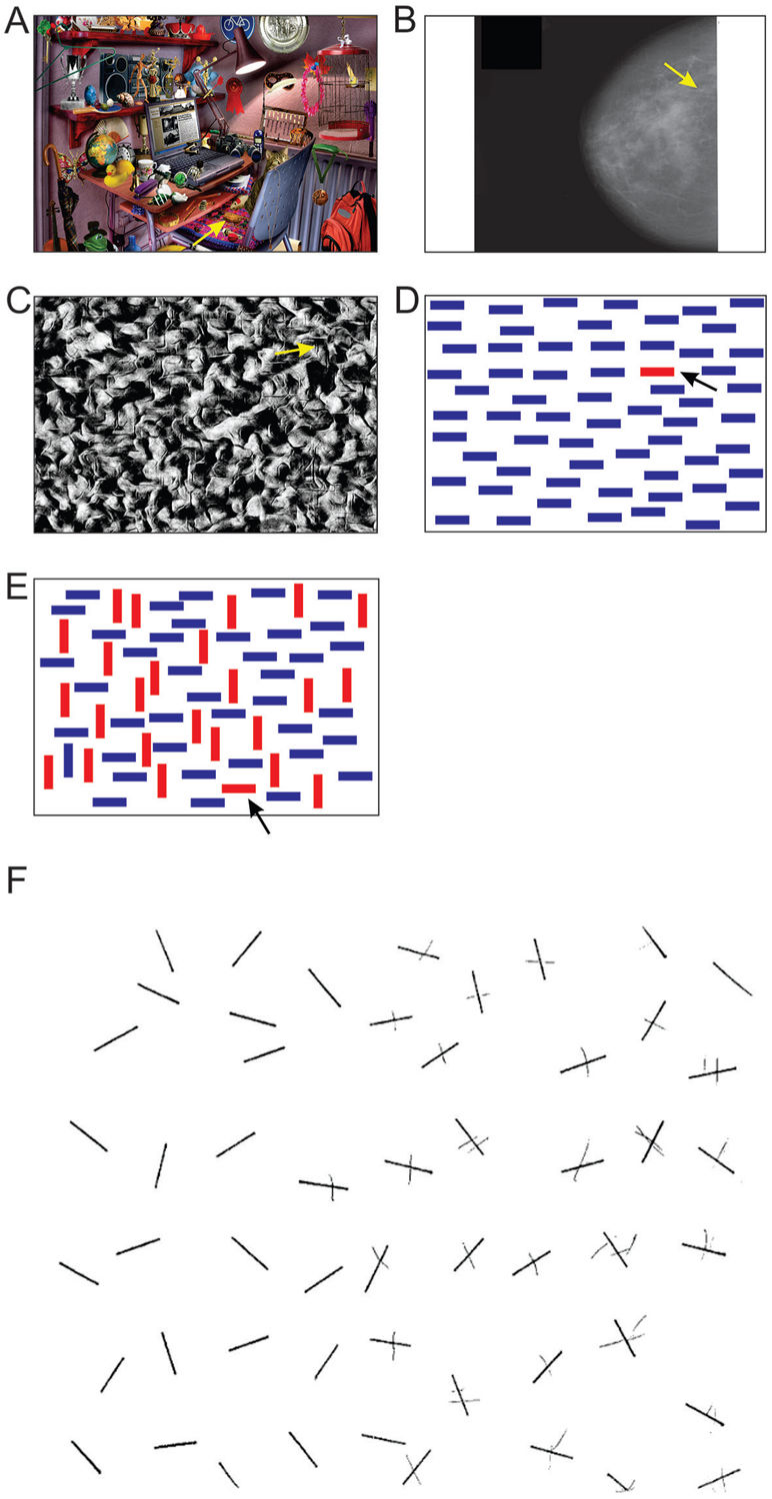
**(A-E)** Answers for the search tasks in Figures 1A-E. The search target in in each panel is indicated by the arrow. **(F)** A task completed by a patient with left hemineglect, caused by damage to the right half of the brain. In this test, the patient was asked to search the entire image and cross out any lines found anywhere in the image. This patient failed to find any lines on the left side of the image because of the damage to the right side of the brain [5].

If various brain regions specialized in various parts of the visual search process, as we described above, it makes sense that when one or more of these brain regions are damaged (due to stroke, trauma, tumor, etc.), visual search is also affected. This is indeed the case [5]. For instance, patients with damage to certain regions of the right half of the brain fail to find objects in the left half of the image, a phenomenon known as “hemineglect” (Figure 3F). This is because the right half of the brain handles information from the left side of the image. Interestingly, damage to the same regions in the left half of the brain does not result in any hemineglect, for reasons that remain largely unclear.

## SUMMARY

Visual search is a large part of how we see in the real world. We understand a good deal about why some objects of interest are easier to find than others. But much remains to be discovered about how the brain works during visual search. Still, it is quite clear that most parts of the brain that play a role in vision also participate in visual search, probably because visual search is such a big part of vision. Thus, damage to some parts of the brain can make visual search more difficult. In the future, computers that can perform successful visual search—such as those that can better detect things like enemies in the battlefield, cancer in X-rays, or potential hazards in airport baggage—are likely to be a bigger part of our lives.

## VISUAL SEARCH

Task that involves actively scanning an environment for a particular object or feature.

## ODD-MAN-OUT

The object that does not belong.

## ATTENTION

Process of concentrating on a specific aspect of information.

## fMRI (FUNCTIONAL MAGNETIC RESONANCE IMAGING)

Imaging that measures brain activity by detecting changes in blood flow.
